# Targeting DUBs to degrade oncogenic proteins

**DOI:** 10.1038/s41416-020-0728-7

**Published:** 2020-02-04

**Authors:** Anjali Cremer, Kimberly Stegmaier

**Affiliations:** 1 Department of Pediatric Oncology, Dana-Farber Cancer Institute and Boston Children’s Hospital, Harvard Medical School, Boston, MA 02215 USA; 2 University Hospital Frankfurt, Department of Hematology/Oncology, Frankfurt/Main, Germany

**Keywords:** Drug development, Acute myeloid leukaemia

## Abstract

Targeted protein degradation has emerged as a strategy in cancer therapy. Yang et al. discovered that HBX19818, an inhibitor of the deubiquitinase (DUB) USP10, leads to the dual degradation of spleen tyrosine kinase (SYK) and FLT3, resulting in death of AML cells.

## Main

After several decades of a stalemate in the treatment of acute myeloid leukaemia (AML), the last 2 years have witnessed the approval of numerous new targeted drugs for patients with AML. In some cases, mutations in the target (e.g. *FLT3* [fms-related tyrosine kinase 3] and *IDH1/2* [isocitrate dehydrogenase {NADP+}]) predict response to the relevant targeted inhibitor. In other cases, such as with the BCL2 inhibitor venetoclax, mutations in the target itself are not a major predictor of response. In either situation, however, a major challenge moving forward will be to circumvent the resistance that will emerge under the selective pressure of these drugs, as well as to expand the armamentarium of targeted agents for patients with AML.

In this issue of the *British Journal of Cancer*, Yang et al. focus on protein degradation as an approach to targeted therapy.^1^ Specifically, they discover an approach to the degradation of FLT3 and spleen tyrosine kinase (SYK) through the inhibition of USP10 (ubiquitin-specific protease 10), a deubiquitinating enzyme. *FLT3* is recurrently mutated in approximately one-third of AML cases through both internal tandem duplications in the juxta-membrane domain and point mutations in the tyrosine kinase domain. In contrast, SYK is more typically activated through integrin and Fc-receptor signalling^2^ with translocations, such as TEL-SYK, a rare event.^3^ SYK is a cytoplasmic tyrosine kinase, best known for its role in B-cell development, but also demonstrated to play an important role in myeloid signalling. Genetic and pharmacological suppression of SYK have been demonstrated to impair cell growth in vitro and in vivo in AML models.^4,5^ Several biomarkers of response to SYK inhibitors in AML have been reported, including high *HOXA9* (homeobox A9) and *MEIS1* (myeloid ecotropic viral integration site-1) expression and *FLT3* mutations.^6,7^ Indeed, SYK has been reported to activate FLT3 through a direct interaction, a finding corroborated in the current study.^1,7^ In support of these preclinical findings, two orally bioavailable SYK inhibitors, entospletinib and TAK-659, have been investigated in clinical trials in patients with AML alone and in combination with standard chemotherapy. Early responses have been reported, especially in patients with *FLT3*-mutated AML and high *HOXA9*/*MEIS1* expression, such as *MLL* (mixed-lineage leukaemia)-rearranged and *NPM1c* (cytoplasmic nucleophosmin 1) mutant leukaemia.^8,9^

Prior studies report that combination therapy targeting SYK and FLT3 with inhibitors selective for each kinase are synergistic in vitro and improve response in vivo compared with each inhibitor alone in *FLT3*-mutated AML.^7,10^ An alternative strategy, however, would be to deploy a small molecule kinase inhibitor that simultaneously targets both, such as the compound TAK-659. Yang et al.^1^ discovered another parsimonious strategy—to degrade both SYK and FLT3 with a single molecule, that of a deubiquitinase (DUB) inhibitor.

**Fig. 1:**
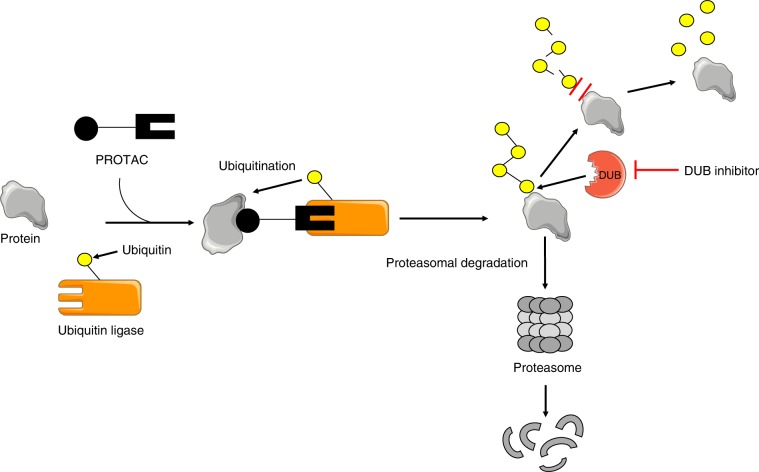
Targeted protein degradation. Two approaches to targeted protein degradation through activation of proteasomal-mediated degradation are depicted: the use of a PROTAC (proteolysis-targeting chimera) or DUB (deubiquitinase) inhibitor.

The notion of targeted protein degradation as a cancer therapy has gained momentum in recent years. For example, arsenic trioxide and thalidomide derivatives, drugs reported to have clinical activity prior to mechanistic insight, were later demonstrated to work via protein degradation. Arsenic trioxide targets the PML-RARα (promyelocytic leukaemia-retinoic acid receptor α) fusion protein in acute promyelocytic leukaemia (APL) for proteasomal-mediated degradation.^11^ In multiple myeloma, thalidomide derivatives act as “natural glues” by binding to the E3 ligase cereblon (CRBN) and by redirecting its substrate specificity to bind and degrade IKZF1 (IKAROS family zinc finger 1 and IKZF3, essential transcription factors in this disease.^12^ A second method to degrade proteins uses proteolysis-targeting chimeras (PROTACs) (Fig. 1).^13^ Here, a heterobifunctional molecule can be engineered that is comprised of two distinct chemical moieties: a small molecule that can bind to the target protein of interest bridged by a chemical linker to a second small molecule that binds to an E3 ligase protein, such as CRBN or VHL (Von Hippel Lindau). The PROTAC brings the target and the E3 ligase machinery in close proximity, resulting in ubiquitination of the target protein followed by proteasome-mediated degradation. Such approaches hold promise for drugging difficult cancer targets, such as transcription factor fusions.

Yang et al.^1^ focus on a less explored approach to targeted degradation in cancer—the inhibition of DUBs (Fig. 1). There are two main classes of DUBs: cysteine proteases and metalloproteases, with the ubiquitin-specific proteases (USPs) a subfamily of the cysteine proteases. DUBs cleave the peptide or isopeptide bond between ubiquitin and the substrate protein. One DUB inhibitor under preclinical evaluation is HBX19818, which inhibits the activity of USP7 and USP10.^14^ Yang et al. provide compelling evidence that SYK can be degraded by inhibition of USP10 leading to cell death in AML cells driven by activated SYK or mutant FLT3.^1^ The same group had shown previously that USP10 is also the major deubiquitinating enzyme for FLT3.^15^ USP10 inhibition thus leads to dual degradation of FLT3 and SYK, which is beneficial; the combined inhibition of FLT3 and SYK by small molecules is more efficacious than inhibition with either an FLT3 or SYK inhibitor alone in AML.^7,10^

There are several advantages to a degrader approach as a cancer therapy.^13^ In principal, there is less need for high systemic drug exposures to maintain sufficient target inhibition in vivo, resulting in fewer off-target effects. Second, degradation strategies may enable the targeting of proteins that have been considered “undruggable”, such as scaffolding proteins and transcription factors. Degradation of kinases might also offer additional therapeutic benefit by eliminating both the enzymatic activity and any scaffolding function that the kinase contributes. Moreover, degraders have the ability to counteract the increased target protein expression that frequently accompanies inhibition of protein function. Finally, as the mechanism of resistance to kinases inhibitors is often a new gatekeeper mutation in the kinase itself, degradation eliminates this possibility.

The findings of Yang et al. prompt the more systematic study of the mechanisms by which cancer-associated protein stability is regulated.^1^ With the possibility now of genome-scale CRISPR-Cas9 screens, one can envision systematic approaches to identify the DUBs/E3 ligases that regulate critical cancer targets. The specific E3 ligases and DUBs involved in the regulation of challenging protein targets might be more easily inhibited by small molecules because they possess enzymatic activity. Moreover, such genome-scale screening approaches can be utilised to anticipate resistance mechanisms to molecules such as HBX19818. Looking to the future, it will be important to test optimised DUB inhibitors in vivo and to dissect the differences in activity of enzymatic inhibitors versus degraders of targets such as SYK and FLT3 alone and in combination.

## Data Availability

N/A
